# O1-3 Time spent cycling, running, walking, standing, sitting, and lying: a cross-sectional analysis of accelerometer-data from 930 Danes aged 15-94 in the Moving Denmark study

**DOI:** 10.1093/eurpub/ckac094.003

**Published:** 2022-08-29

**Authors:** Koch Sofie, Jasper Schipperijn, Lars Breum Christiansen

**Affiliations:** University of Southern Denmark, Odense, Denmark

**Keywords:** Physical activity behavior, Accelerometer, Adolescents, Adults, Cross-sectional study

## Abstract

**Background:**

Physical activity is essential for public health. Newer device-based measures can identify different activity behaviors, which is useful to focus future health promotion initiatives. The objectives of this study were to describe time spend cycling, running, walking, standing, sitting, and lying during a regular day using accelerometers, and to describe differences between age-groups and gender.

**Methods:**

In the Moving Denmark study a representative sample of 400.000 Danes were invited to a national survey. A subsample of 1525 respondents gave consent to wear an accelerometer (Axivity AX3 attached to the right thigh; 24 h/day, 7 consecutive days) of which 930 fulfilled the inclusion criteria (min. 3 valid weekdays and 1 valid weekend day with data). Daily time spent biking, running, walking, standing, sitting, and lying were derived using the method proposed by Skotte et al. (2014) using 1-s epochs, and differences between age-groups (15-25, 26-45, 46-65, +66 y/o) and gender were investigated using Kruskal-Wallis rank sum test.

**Results:**

Among those cycling (39.2%) and those running (67.9%), the mean time per day was 8.4 minutes for cycling and 2.8 minutes for running. The mean time walking, standing, sitting, and lying was 93.5, 183.2, 572.1, and 542.2 min/day, respectively. No significant difference was observed between age groups on biking, but time spend biking was highest among men. Average time spend running and walking was highest among the youngest participants (15-25 y/o), but with no differences observed on gender. No difference between age groups were found for standing, but the average time spend standing was significantly higher for women compared to men. Average sitting time was significantly higher among the older participants (+66 y/o), whereas time spend lying was highest among the youngest participants, but with no differences observed on gender.

**Conclusions:**

The study show that at least 67.9% of the sample are active through either biking, running, or both, but also that the average daily minutes are limited. Men accumulate in average more minutes of biking, and women more minutes of standing. The younger participants had more minutes of walking, running, and lying, while the older participants more often were sitting.

